# Detection of High-Grade Prostate Cancer With a Super High B-value (4000 s/mm2) in Diffusion-Weighted Imaging Sequences by Magnetic Resonance Imaging

**DOI:** 10.7759/cureus.22807

**Published:** 2022-03-03

**Authors:** Maria Jose Acosta-Falomir, Juan Carlos Angulo-Lozano, Luisa Fernanda Sanchez-Musi, Danny Soria Céspedes, Yeni Fernández de Lara Barrera

**Affiliations:** 1 Radiology, The American British Cowdray Medical Center, Mexico City, MEX; 2 School of Medicine, Universidad Anáhuac México, Mexico City, MEX; 3 Urology, Hospital General de México, Mexico City, MEX; 4 School of Medicine, Universidad Anáhuac México, Huixquilucan, MEX; 5 Pathology, The American British Cowdray Medical Center, Mexico City, MEX; 6 Radiology and Molecular Imaging, The American British Cowdray Medical Center, Mexico City, MEX

**Keywords:** magnetic resonance imaging, prostate cancer (pca), uroradiology, mri diffusion-weighted image, pi-rads

## Abstract

Introduction: High-grade adenocarcinoma of the prostate tends to have denser glandular structures and a prominent desmoplastic reaction, which could be detected by magnetic resonance imaging (MRI) with a super-high b-value in diffusion-weighted imaging (DWI) sequence, to differentiate it from low-grade carcinomas.

Objective: To evaluate the diagnostic validity of the diffusion sequence with values ​​of b4000 s/mm2 for the diagnosis of high-grade prostate cancer (Gleason score ≥ 7).

Materials and methods: It is a retrospective analytical study of male patients who have undergone a prostate biopsy and count with a prostate MRI with a DWI sequence of a super-high b-value (4000 s/mm2).

Results: The sensitivity of the diffusion sequence with b4000 s/mm2 values ​​to classify as positive for prostate cancer was 57.14% as compared to biopsy. The specificity of the diffusion sequence with b4000 s/mm2 values ​​classifying patients with prostate carcinoma as negative was 84.62%. The probability that the diffusion sequence with b4000 s/mm2 values ​​classifies patients with prostate cancer was 80%. The probability that the diffusion sequence with b4000 s/mm2 values ​​does not classify patients with prostate cancer was 64.71%. The proportion of patients adequately classified with prostate cancer using the diffusion sequence with b4000 s/mm2 values ​​was 70.37%.

Conclusions: The study shows that using the diffusion sequence with values of b4000 s/mm2 is an optimal value that serves as a tool to be able to decant those high-risk carcinomas with those of low risk; however, it is not a definitive method of diagnosis that could replace the performance of a biopsy. Since the study sample was limited, these results cannot be interpreted as reliable for diagnosing high-grade prostate cancer and should encourage future studies on a larger scale population to obtain significant evidence for a non-invasive diagnostic tool with a better cost-benefit for the patient.

## Introduction

Prostate cancer (PC) is the second most common cancer in the male population and the fourth in the global population. A prostate biopsy is the main way to diagnose PC. However, it could have some morbidities. PC represents 14.1% of all cancers that affect men, and it is only below lung cancer (14.3%). In the USA, it is the most prevalent cancer in men (14.2%), with 1,441,001 cases diagnosed in 2020 and a mortality rate of 6.9% (382,761), similar to the global described ones (6.8%). Of cases, 70% occur in developed countries. It is the sixth cancer with the highest mortality in men, with a rate of 7.7 deaths per 100,000 men [1].

The clinical manifestations are often absent at the time of diagnosis. The clinical behavior of PC varies from asymptomatic, microscopic, well-differentiated detected tumor that may never be clinically significant, high-grade asymptomatic, or critically symptomatic aggressive high-grade cancer causing metastasis, morbidity, and death. At diagnosis, 78% of patients have localized cancer, 12% have regional lymph node involvement, and 6% have distant metastases [2]. A biopsy may show PC or precancerous or benign findings. If the biopsy indicates PC, the architectural features of the cells in the biopsy tissue are used to classify a Gleason grade that correlates closely with clinical behavior. The Gleason grade is critical in determining the treatment approach [3]. Using the standard 12-sample biopsy, less than 1% of the prostate gland is sampled. This limited sampling explains why a random biopsy of 12 samples may miss the tumor in at least 20% of cases and why the Gleason grade may be underestimated in another 20-30% of cases. High-grade adenocarcinoma of the prostate tends to have denser glandular structures and a prominent desmoplastic reaction, which may be better detected by magnetic resonance imaging (MRI). An image-guided biopsy will detect 10-15% more PCs than the standard 12-sample biopsy and is particularly effective at detecting clinically significant lesions. We proceed with a biopsy if life expectancy is at least 10 years and the prostate-specific antigen (PSA) rises above the range for the patient's age cohort, or the PSA has increased more than 0.75 ng/mL in one year, or there is an abnormality on palpation during a digital rectal examination [4].

Prostatic adenocarcinoma can be diagnosed by the presence of small infiltrating glands with prominent nucleoli. Architecturally, malignant cells form glands that are usually smaller than benign glands (acini or ducts), and tumor cells tend to infiltrate the glandular tissue in a disorganized matter. In less differentiated tumors, the glandular pattern is irregular, less organized, or even absent, and tumor cells tend to grow as cords nests, most often in cribriform patterns. Tumor cells often show nuclear enlargement, and irregularity, hyperchromasia, and prominent nucleoli can be seen in most cases. Intraluminal crystalloids, amorphous secretion, or blue-stained mucins are frequently present in malignant glands but infrequently found in benign glands. The Gleason score is derived by adding the numerical values ​​for the two most prevalent patterns of differentiation (a primary grade and a secondary grade). For example, if a biopsy consists predominantly of grade 3 and secondary grade 4 disease, the combined score is "3 + 4" or 7. The range of Gleason scores in prostate biopsies is from Gleason score 6 to 10 [3]. This article aims to assess the value of diffusion-weighted imaging (DWI) sequences by MRI for the detection of high-grade PC.

## Materials and methods

Patient selection

This retrospective analytical study included 27 male patients who had undergone a prostate biopsy and had a prostate MRI with the DWI sequence with a super-high b-value (4000 s/mm2).

Inclusion criteria included patients who had a prostate biopsy performed and who had an MRI of their prostate with DWI with a super-high b-value of 4000 s/mm2. Exclusion criteria included a result of a negative prostate biopsy for cancer and patients who do not have MRI studies performed at a medical center.

Radiological evaluation

Prostate biopsies carried out from August to December of 2021 were reviewed. MRI studies performed in the hospital from August to December of 2021 were examined to confirm that the study was done with the diffusion sequence with a b-value (4000 s/mm2) and it approved and reported the type of lesion from the diffusion sequence. MRI was performed on Siemens 3 Tesla equipment (Siemens Healthineers, Erlangen, Germany). Two people reviewed each study. The first interpretation was made by a radiologist studying a high specialty in MRI or by an assigned doctor. The final decision was made by a certified radiologist other than the one for the first interpretation.

Statistical evaluation

Statistical analysis and methodology were reviewed by an expert on biostatistics from the department of public health of the same medical center. Images were evaluated on high-resolution monitors and classified according to the Prostate Imaging-Reporting and Data System (PI-RADS) version 2.1 system, a structured reported measure for multiparametric prostate MRI when evaluating suspected PC using different MRI sequences (T2-weighted (T2W), DWI, and dynamic contrast-enhanced (DCE)) [5]. An Excel (Microsoft Corporation, Redmond, WA) database and an exploratory analysis were carried out to verify the quality of the records. Subsequently, a univariate analysis was made to explore normality in the case of quantitative variables and review data out of range. For the variables measured on a ratio scale, the average and standard deviation were used; in the case of variables measured on an ordinal or nominal scale, they were reported in frequency and percentage. Inferential analysis was performed to determine the validity of the b4000 s/mm2 diffusion sequence through sensitivity, specificity, positive predictive value, negative predictive value, and the receiver operating characteristic (ROC) curve. Data were analyzed using Stata version 15 (StataCorp LLC, College Station, TX) software. ANOVA test was used to evaluate the differences between the means of PI-RADS and the apparent diffusion coefficient (ADC) values groups. A Student's t-test was performed to assess the mean differences of the positive and negative DWI sequence with a b-value greater than 4000 s/mm2 comparing the ADC mean value. Fisher's exact test was used to assess the association between the histologic Gleason score lower than 6 or greater than 7 with a positive or negative result on the DWI sequence of b4000 s/mm2, the association between PI-RADS score with a score of 3 or greater than 3 with the DWI sequence of b4000 s/mm2, and the association of the prostatic zone involved with cancer and the DWI sequence of b4000 s/mm2. For the analysis of sensitivity, specificity, positive predictive value, and negative predictive value, the chi-square test and the ROC curve were used.

## Results

Regarding the classification of injuries according to PI-RADS version 2.1, it was found that 22.22% of patients obtained a PI-RADS 3 classification, 55.56% were classified as PI-RADS 4, and 22.22% were classified as PI-RADS 5. In the anatomical location of the lesions, it was observed that 44.44% of patients were in the peripheral zone while 55.56% were in the transitional area. Regarding post-gadolinium enhancement, 44.44% of patients presented early enhancement. When evaluating the diffusion sequence of b4000 values, it was found that 37.04% of patients showed restriction (Table 1).

**Table 1:** Image characteristics and classification according to PI-RADS version 2.1 and Gleason scale. PI-RADS: Prostate Imaging-Reporting and Data System.

Variable	Frequency	Percentage
PI-RADS		
3	6	22.22%
4	15	55.56%
5	6	22.22%
Injury by area		
Peripheral	12	44.44%
Transitional	15	55.56%
Early enhancement of the lesion		
Yes	12	44.44%
No	15	55.56%
Evaluated with b4000 s/mm2		
Positive	10	37.04%
Negative	17	62.96%
Gleason scale		
0 (benign)	12	44.44%
6	1	3.70%
7	11	40.74%
8	2	7.41%
9	1	3.70%

In the histopathological classification based on the Gleason scale, it was found that 44.44% of patients obtained a score of 0 (simple atrophy, fibroglandular hyperplasia, and no carcinoma), 3.70% received a score of 6 (3 + 3), 40.74% obtained a score of 7 (4 + 3, 3 + 4), 7.41% received a score of 8 (4 + 4), and 3.70% received a score of 9 (5 + 4) (Table 1).

On the ADC value and the PI-RADS classification, it was found that those who were classified with PI-RADS 3 have an average ADC value of 964 m2/s (147.95), those who were classified with PI-RADS 4 obtained an average ADC value of 681.73 m2/s (217.15), and those who got a PI-RADS score of 5 had an average ADC value of 680 m2/s (81.85). A statistically significant difference (0.0103) was observed.

Regarding the diffusion variable of patients with values ​​of b4000 s/mm2 and who showed restriction, the patients obtained an average ADC value of 675.1 m2/s (110.24), and those who did not show restriction obtained an average ADC value of 784.82 m2/s (248.83). No significant difference was observed (0.1006).

Based on the diffusion sequence with b4000 s/mm2 and the score with the Gleason scale, it was shown that 20% of patients who showed a lesion with restriction obtained a score less than or equal to 6, while 80% obtained a score greater than or equal to 7; likewise, the lesions that did not show restriction in this sequence, 64.71% of patients got a score less than 6, while 35.29% obtained a score greater than or equal to 7; finding a significant difference (0.046).

Concerning the diffusion sequence with b4000 s/mm2 and the PI-RADS classification, it was observed that 100% of the lesions evidenced in this sequence showed a PI-RADS greater than or equal to 3. As for those who did not show lesions in this sequence, 35.29% showed a PI-RADS 3, while 64.71% obtained a PI-RADS greater than or equal to 4, with a borderline statistical difference (0.057).

Concerning the b4000 s/mm2 diffusion sequence and the area where the lesion was found, it was found that 60% of the lesions that showed restriction in this sequence were found in the peripheral zone, while 40% were observed in the peripheral area. Of those with no restriction, 35.29% were in the peripheral area and 64.71% were in the transitional area, without finding a statistically significant difference (0.237) (Tables 2, 3).

**Table 2:** Bivariate analysis according to ADC values and their classification in PI-RADS and diffusion sequence of b4000. ^d^ ANOVA test; ^l^ t-test. ADC: apparent diffusion coefficient; PI-RADS: Prostate Imaging-Reporting and Data System.

	ADC	
PI-RADS	Mean/standard deviation	Coefficient
3	964 + 147.95	0.0103^d^
4	681.73 + 217.15	0.0103^d^
5	680.5 + 81.85	0.0103^d^
B4000 diffusion sequence		
Positive	675.1 + 110.24	0.1006^l^
Negative	784.82 + 248.83	0.1006^l^

**Table 3:** Bivariate analysis based on the diffusion sequence of b4000 and categories according to the Gleason scale, PI-RADS category, and prostate zone. ^m^ Fisher's exact test. PI-RADS: Prostate Imaging-Reporting and Data System.

	Positive	Negative	Coefficient
Gleason			
Score less than 6	2 (20.0%)	11 (64.71%)	0.046^m^
Score greater than or equal to 7	8 (80.0%)	6 (35.29%)	0.046^m^
PI-RADS			
Score 3	0 (0%)	6 (35.29%)	0.057^m^
Score greater than 3	10 (100%)	11 (64.71%)	0.057^m^
Zone			
Peripheral	6 (60%)	6 (35.29%)	0.257^m^
Transitional	4 (40%)	11 (64.71%)	0.257^m^

An analysis of sensitivity and specificity was performed, as well as the positive and negative predictive values of the b4000 s/mm2 diffusion sequence were compared with the gold standard, which in this case is the histopathological study.

The sensitivity of the diffusion sequence with b4000 s/mm2 values ​​to classify as positive for PC was 57.14% compared to biopsy. The specificity of the diffusion sequence with b4000 s/mm2 values ​​classifying patients with prostate carcinoma as negative was 84.62%. The probability that the diffusion sequence with b4000 s/mm2 values ​​classifies patients with PC was 80%. The probability that the diffusion sequence with b4000 s/mm2 values ​​does not classify patients with PC was 64.71%. The proportion of patients adequately classified with PC using the diffusion sequence with b4000 s/mm2 values ​​was 70.37% (Tables 4, 5).

**Table 4:** Contingency model for evaluation of the diffusion sequence with values of b4000 and prostate biopsy.

	Prostate biopsy		
Resonance b4000 sequence	Positive	Negative	
Positive	8	2	10
Negative	6	11	17
Total	14	13	27

**Table 5:** Analysis of sensitivity, specificity, PPV, and NPV. ^s ^Chi-square test. PPV: positive predictive value; NPV: negative predictive value; ROC: receiver operating characteristic.

	Sensitivity	Specificity	PPV	NPV	Correct classification
Sequence b4000	57.14%	84.62%	80.00%	64.71%	70.37
Area ROC	70.88%	P-value	0.0340^s^		

**Figure 1:**
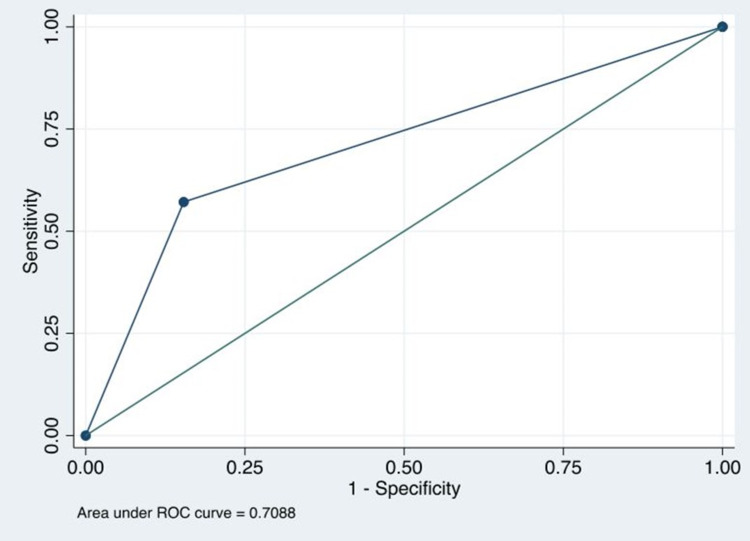
The ROC curve of the DWI sequence with a b-value of 4000 m/sec2 for diagnosis of high-grade prostate cancer. ROC: receiver operating characteristic; DWI: diffusion-weighted imaging.

The area under the curve (ROC) was 70.88%, with a significant difference concerning the biopsy (p = 0.0340) (Figure 1).

## Discussion

DWI plays an essential role in MRI for the study of prostate carcinoma. The binomial of ADC and DWI map sequences are used as an important image marker for the presence of normal and altered prostate tissue. However, an optimal b-value has not been found to lead to the detection of high-risk carcinomas concerning prostate tissue with low risk. In this work, the diagnostic validity of the diffusion sequence with super high values ​​of 4000 s/mm2 was evaluated to distinguish high-risk carcinomas, finding sensitivity and specificity of 57.14 and 84.62%, respectively, with an area under the curve of 70.88 (p = 0.0340), which translates that despite being a test with a normal range, it allows guiding the multi and transdisciplinary team to perform, avoid, or postpone a biopsy. The area under the curve is not decisive, which indicates that this sequence could miss some tumors and false positives and negatives. Although it is an advanced method, it is not a definitive method for diagnosing high-risk carcinomas.

For many years, PC screening was carried out based on PSA levels and physical examination. The use of PSA was mainly for the early diagnosis of PC and questions were asked about how many of these tumors require some intervention since they put the life or well-being of the patient at risk, and how many of them only require close follow-up. Therefore, the criteria for clinically significant cancer were established: a Gleason score of 7 or greater, a tumor with extraprostatic extension (T3a or greater), and a tumor volume in prostatectomy greater than 0.5 ccs. The three main components in pathology to establish the eligibility criteria for the different active surveillance protocols are the number of samples with cancer, the percentage, and the degree [6]. Initially, prostate MRI was used to assess locoregional invasion in patients with a positive biopsy; with technological advances, it has been possible to increase the applications of prostate MRI to detect clinically significant cancer, perform targeted biopsies, and thus reduce associated morbidity and mortality. Due to the considerable inconsistencies in the protocol, interpretation, and reporting of prostate MRI, version 1 of PI-RADS was published in 2012 [7]. In response to rapid progress and awareness of the limitations of version 1 in 2015, version 2 (v2) was released. In 2019, some clarifications were made and it was released as version 2.1 (v2.1) [6,8]. There is a more significant interobserver agreement with the most recent version of PI-RADS [9]. Multiple sequences are performed in the MRI, including DWI. The DWI depends on the microscopic mobility of water. This mobility, classically called Brownian motion, is due to thermal agitation and depends on the cellular environment of the water [10,11]. In biological tissues, however, the diffusion of water is restricted by different components, such as the cellularity of the tissue, the organization of the tissue, the tortuosity of the extracellular space, and the integrity of the cell membranes. The degree of restriction of the water diffusion in biological tissue is inversely related to the cellularity of the tissue and the integrity of cell membranes; the rate of water diffusion is more restricted in tissues with high cell density due to the presence of numerous intact cell membranes [12]. In PC, the loss of luminal and ductal spaces of normal glandular tissue and increased cell density leads to a decrease in water diffusivity [13].

According to the parameters suggested by the PI-RADS, a DWI with a b-value of at least 1400 s/mm2 should be performed to detect clinically significant cancer [6]. Compared with ADC maps alone, the visibility of clinically substantial cancers is sometimes improved on high b-value images, especially those adjacent to or invading the anterior fibromuscular stroma, in a subcapsular location, and at the apex the base of the gland. High b-value images can be obtained in one of two ways: directly by acquiring a high b-value DWI sequence (which requires additional scan time) or by calculating (synthesizing) the high b-value image by extrapolation of the lower acquired value [14,15]. B-value data are used to create the ADC map (potentially less prone to artifacts because it avoids the longer time to echo required to accommodate the intense gradient pulses needed for high b-value acquisitions). As the b-value increases, the signal-to-noise ratio decreases, so the optimal high b-value may depend on the strength of the magnetic field, the software, and the manufacturer [16]. In theory, "brightness through T2" could be overcome by using b-values ​​in the range of 1500-2000 s/mm2 or more; however, the acquisition of very high b-values ​​in clinical practice is complicated by technical problems and very long scan times [17]. Therefore, there is currently no widely accepted optimal “high b-value” beyond the requirement for a set of DWI images with a b-value ≥ 1400 s/mm2. However, DWI with higher b-value yields more significant suppression of benign tissue and thus potentially better tumor visualization. Direct acquisition of such b-values ​​is technically challenging due to issues related to the reduced signal-to-signal ratio: noise and an increase in anatomical distortion and resulting artifacts [18]. A study was carried out with a b-value of 1600 s/mm2, identifying lesions with an increase in the signal in the diffusion sequence, of which 71% corresponded to PC, calculating a sensitivity of 75%, a specificity of 82%, a value positive predictive of 66% and negative of 87% [6]. A meta-analysis determined that the sensitivity reported for high b-values ​​of 1400-2000 s/mm2 is 59-99%, and the specificity is 72-98% [19,20]. In another study, a higher sensitivity was observed to detect tumors with b-values ​​between 1500 and 3000 s/mm2 for the first observer and between 1500 and 2500 s/mm2 for the second observer. The highest sensitivity was achieved for Gleason score ≥ 7 tumors with b-values ​​between 1500 and 2000 s/mm2 compared to the ADC map. They concluded that visualization in DWI with a b-value between 3000 and 5000 s/mm2 is compromised by the difficulty in locating the lesion, which is why they recommend using a b-value between 1500 and 2500 s/mm2 [18]. Agarwal et al. determined that there is no statistically significant difference between the area under the curve of the acquired DWI and any of the calculated DWI; they also found that the highest area under the curve is 0.74 in acquired DWI with a b-value of 1600 s/mm2 for the detection of intermediate-high grade PC, and with the DWI calculated, the area under the highest curve was obtained with a b-value of 2125 s/mm2, mounting a minimum decrease to a b-value of 4000 s/mm2 [21]. Jendoubi et al. conducted a study to compare the usefulness of acquiring DWI sequences with a high b-value or performing calculated DWI with two low b-values ​​and determined that with a calculated b-value of 2000 s/mm2 and 2500 s/mm2, there is better image quality, greater background tissue suppression, greater anatomical clarity, and less distortion [21]. In another study, they observed that the DWI with a b-value of 2000 s/mm2, when compared with a b-value of 1000 s/mm2, is more beneficial for less experienced radiologists for detecting PC in the peripheral zone, without finding a statistically significant difference for the transitional area [2]. Feuerlein et al. reported similar findings [17]. Despite having so many studies, a consensus has not yet been reached on the correct b-value to diagnose clinically significant cancer. The present study had certain limitations. The sample size was limited, which could introduce a selection bias; that is why results should be taken with caution and the nature of the study itself, so the study results could not be extrapolated because of the external validity.

## Conclusions

The study shows that using the diffusion sequence with values ​​of b4000 s/mm2 is an optimal value that serves as a tool to be able to decant those high-risk carcinomas with those of low risk; however, it is not a definitive method of diagnosis that could replace the performance of a biopsy. Since the study sample was limited, these results cannot be interpreted as reliable for diagnosing high-grade PC and should encourage future studies on a larger scale population to obtain significant evidence for a non-invasive diagnostic tool with a better cost-benefit for the patient.
